# Patient Satisfaction With Medications for Opioid Use Disorder Treatment via Telemedicine: Brief Literature Review and Development of a New Assessment

**DOI:** 10.3389/fpubh.2020.557275

**Published:** 2021-01-21

**Authors:** Thomas O. Cole, Darlene Robinson, Andrea Kelley-Freeman, Devang Gandhi, Aaron D. Greenblatt, Eric Weintraub, Annabelle M. Belcher

**Affiliations:** ^1^Division of Addiction Research and Treatment, Department of Psychiatry, University of Maryland School of Medicine, Baltimore, MD, United States; ^2^Life's Energy Wellness Center, Inc., 8737 Brooks Drive, Easton, MD, United States

**Keywords:** patient satisfaction, telemedicine, opioid use disorder, medication assisted treatment, rural, telebehavioral health, buprenorphine

## Abstract

Telemedicine is increasingly being used to treat patients with opioid use disorder (OUD). It has particular value in rural areas of the United States impacted by the opioid crisis as these areas have a shortage of trained addiction medicine providers. Patient satisfaction significantly impacts positive clinical outcomes in OUD treatment and thus is of great clinical interest. Yet little is known regarding patient satisfaction with the increasingly important platform of telemedicine-delivered medications for opioid use disorder (tMOUD). The goal of this review is to provide a summary of the existing literature regarding patient satisfaction with tMOUD. We also submit a novel survey based on an existing framework designed to assess tMOUD satisfaction, and present pilot data (*N* = 14) acquired from patients engaged in rural tMOUD care. Telemedicine provides a feasible method for delivering MOUD in rural areas, and our survey provides a useful assessment to measure patient satisfaction with tMOUD. In light of the pressing need for innovative and technology-driven solutions to the opioid epidemic (especially in light of the COVID-19 pandemic), future research should focus on the development and refinement of tools to assess the important implementation goal of patient satisfaction.

## Introduction

The United States is facing an opioid crisis that has little precedent. Once a problem constrained primarily to urban areas, the past two decades have witnessed a significant geographic shift, with rural communities experiencing increases both in non-medical opioid use and in the number of opioid overdose deaths. Rural areas of the United States have been disproportionately impacted by this crisis, with higher reported rates of non-medical opioid use and overdose compared to urban areas (1, 2). From a treatment capacity perspective, rural areas often lack providers who have met the training and registration requirements to prescribe buprenorphine for treatment of opioid use disorder (OUD). This results in a lack of access to medications for opioid use disorder (MOUD), rendering these communities poorly equipped to treat individuals with OUD (3–5).

The Centers for Medicare and Medicaid Services (CMS) is the Federal agency responsible for administering payments for medical services to seniors and low-income individuals in the United States. CMS defines telemedicine as two-way, real time interactive communication between patients and physicians or practitioners at a distant site for the purpose of improving patients' health (6). Telemedicine offers a viable solution to increase access to MOUD given its potential to fill gaps in provider access. The utility and value of telemedicine has been heightened by the emergence of COVID-19, as the need for remote, contactless methods of health care delivery is now crucial. Patient satisfaction is an important factor in the assurance of validation and acceptance of this emerging care medium. This variable is especially critical in the treatment of OUD, as previous reports have shown that satisfaction has a direct impact on treatment outcomes (7–10).

Although patient satisfaction has been accorded a great deal of attention in the frame of telehealth- and telemedicine-based care modalities, the literature is almost non-existent for telemedicine-delivered MOUD (tMOUD). The goal of this review is to summarize this literature and to present pilot data for a survey that we have created to assess patient satisfaction with tMOUD.

## Literature Review

### The Rural OUD Problem

The past two decades have witnessed an explosive opioid crisis in the United States. With the highest rates of opioid prescribing (11), as well as the highest per capita opioid-related death rates, rural areas of the country have been hardest hit by the crisis (12). The rural OUD problem is further exacerbated by a lack of available treatment options. Although behavioral and psychosocial treatment approaches for OUD occupy a rightful space and offer benefits for many people (13), treatment with medications that have action at opioid receptors is the most evidence-based method of treatment for OUD (14–16). But numerous barriers preclude access to MOUD in rural areas, the most significant of which is the lack of specialized providers able to prescribe MOUD (17). Rural OUD treatment seekers must often travel long distances and times— a burden that adds to the challenge of accessing and retaining individuals in care (12, 18, 19).

Socio-cultural factors are also believed to play a role in the disparities between rural and urban communities. Several factors have been identified that explain the disproportionate use of opioids in rural areas, many of which revolve around the fact that rural denizens tend to be employed with labor intensive work—work which poses an increased risk of occupational injury and a cultural acceptance of opioid use to manage pain (20, 21). Rural areas also tend to be comprised of older populations, who often have a greater frequency of chronic pain and thus, higher rates of opioid prescribing (21). Additionally, a lack of economic opportunity and closer-knit social and family networks that facilitate the distribution of opioids has compounded the prevalence of OUD in rural areas (21, 22). Regardless of the causes, there is a critical need to close the OUD treatment gap in rural areas of the United States, for which telemedicine shows promise.

### Telemedicine for the Treatment of OUD

Telemedicine platforms have been utilized in the delivery of psychiatric care that incorporates psychiatric evaluations, therapy sessions, patient education, and medication management. Evaluations of telemedicine include several outcome measures; among these are feasibility, validity, reliability, satisfaction, cost, and clinical outcomes. Previous research has shown that telemedicine is effective in delivering treatment for mental health disorders, such as PTSD, depression, and ADHD (23), findings which paved the way for telemedicine expansion to OUD treatment. Telemedicine is being used increasingly for the treatment of OUD, and has recently been raised to national awareness in light of new federal and state regulations governing MOUD treatment during the COVID-19 pandemic (24). Along with others, our group has shown that treating OUD via telemedicine produces clinical outcomes (specifically, retention and illicit substance use) that are not significantly different from face-to-face interactions (25–27).

The University of Maryland School of Medicine Division of Addiction Research and Treatment (DART) has been at the forefront of delivering MOUD via telemedicine to rural areas of Maryland's eastern-shore and western Appalachian communities. Since August 2015, DART has partnered with intensive outpatient programs (IOP) as well as behavioral treatment programs in outlying, rural areas of the state (Caroline, Talbot and Dorchester Counties on the eastern-shore, and rural areas of Garrett and Washington Counties in western Maryland) to provide tMOUD for opioid-dependent patients. These counties have been significantly impacted by the opioid epidemic, with trends that show little sign of reversing. To illustrate, although the State of Maryland's opioid overdose rate decreased between 2018 and 2019, each of these counties saw either an increase or a relative flat line in the number of opioid overdose deaths (28). To date, our clinicians have treated over 450 patients with the combination of telemedicine and remote buprenorphine prescription, with planned expansions of our services to reach incarcerated populations (29).

Telemedicine-based treatment for OUD can take many forms, but briefly, patients are screened by trained substance use disorder (SUD) counselors at a partnering program. Patients who satisfy criteria for a diagnosis of OUD and who consent to treatment with telemedicine are referred to a telemedicine provider (either an addiction medicine physician or a federally waivered clinician). Throughout the course of treatment, patient encounters occur *via* a HIPAA-compliant synchronized live video-feed (25, 26).

The COVID-19 pandemic has changed the environment of how MOUD treatment is structured. Pre-COVID, in compliance with the Ryan Haight Online Pharmacy Protection Act of 2008, Drug Enforcement Agency (DEA) regulations required that an in-person medical evaluation be conducted by a prescribing clinician for the initial screening of patients (30). Shortly following federal emergency declarations in March 2020, as with other agencies (SAMHSA and the Centers for Medicare & Medicaid Services [CMS]), the DEA eased regulations to facilitate OUD treatment access compatible with public health recommendations for social distancing. The most relevant changes for MOUD treatment included the elimination of the in-person health and physical examination for buprenorphine induction and the allowance (and coverage) of virtual (phone or video) counseling services (31, 32). Although these changes are in effect only for the duration of the public health emergency, these modifications have commanded attention to telemedicine, further heightening the need for data on patient acceptance of this mode of healthcare.

### SUD and Patient Satisfaction

Patient satisfaction has been assessed in SUD-treated populations, and is associated with several important treatment outcomes, including reduction in substance use and greater retention, compliance, and patient engagement. In one of the earliest studies assessing patient satisfaction with SUD treatment, Carlson & Gabriel (33) found that among those starting an outpatient or residential SUD program, service use, satisfaction with access and satisfaction with treatment effectiveness was significantly associated with 1-year abstinence. Hser et al. (10) found that among those starting a drug-free outpatient or residential treatment program, patient satisfaction and service utilization had a positive relationship with treatment completion or longer treatment retention. Consistent with these findings, Kelly et al. (9) found a positive association between patient satisfaction and treatment retention. Specifically, methadone patients who were more satisfied with their treatment program and counselor were more likely to remain in treatment and were less likely to have self-reported cocaine and heroin use, positive urine toxicology results for cocaine and heroin, and involvement in illegal activity (9). In a systematic review of the literature exploring the link between patient satisfaction and treatment outcomes, Barbosa et al. (8) concluded that although there is broad support for an association between patient satisfaction and treatment compliance, adherence, and persistence, there is a pressing need for additional research. Lastly, Hawkins et al. (7) evaluated the acceptance and satisfaction of a Care Management Model (CMM) among a SUD treatment patient population and found that “trust” within patient-provider relationships was a contributing factor in patient engagement with CMM services. Collectively, these findings highlight the importance of focusing attention on patient satisfaction, as a variable that may have profound impact on several aspects of treatment outcomes.

### Patient Satisfaction With Telemedicine

Although it is clear that satisfaction is an important factor in clinical outcomes for SUDs and OUD, little work has been done to evaluate patient satisfaction within the context of tMOUD. In a review of telemedicine-delivered treatment interventions for SUDs, Lin et al. (34) highlighted the finding that no article specifically examined patient satisfaction with MOUD, nor administered a patient satisfaction survey (26, 27, 35). The remaining articles included in that systematic review examined tobacco cessation and psychotherapy for alcohol use disorder and OUD. Eight out of the ten remaining articles administered satisfaction surveys, however, these surveys pertained to satisfaction with counseling services and experiences. Our own search for a literature on patient satisfaction with tMOUD underscored this gap; to our knowledge, no report has evaluated patient satisfaction with the emerging clinical medium of telemedicine with MOUD for the treatment of OUD.

Below, we report on the development and proof of concept implementation of a novel patient satisfaction survey specific to tMOUD. We based our survey on characteristics of previously published patient satisfaction surveys, utilizing the following thematic categories to frame the patient's subjective experience: (i) *communication; (ii) privacy; (iii) patient perceptions; and (iv) technology utilization*. These categories represent areas on which to assess general satisfaction, and have been implemented in other arenas of research, including the delivery of general mental health services, telemedicine for alcohol use disorder, and telemedicine consultations for mental health disorders (36–42). In addition to those four thematic categories, our survey also includes a thematic category to capture *(v) treatment access*, pertinent to understanding patient satisfaction with the availability of care.

## Development of a New Survey for Patient Satisfaction With Telemedicine With MOUD

### Patient Satisfaction Survey for Telemedicine With MOUD

Our telemedicine with MOUD patient satisfaction assessment is a 16-item survey with questions organized according to five thematic categories designed to evaluate satisfaction. See Appendix-1 for full instrument questions.

Using thematic categories interpreted from previous published literature (36–42), we created a survey that assessed satisfaction with: *(i) communication; (ii) privacy; (iii) patient perceptions; (iv) technology utilization; and (v) treatment access*. We describe each of these briefly. The first category, that of *communication* (Questions 1, 5, 6, 7, and 15) relates to the exchange of information between the patient and telemedicine doctor. This category captures aspects of the therapeutic relationship and patient participation in care decisions– important aspects of the therapeutic encounter. *Privacy* (Question 4) is relevant to the extent to which participants felt that their communication with the telemedicine doctor was confidential (i.e., no other patients or staff members could hear their conversation with the telemedicine doctor). *Patient perception* (Questions 8–11, 14) relates to a patient's acceptance of the telemedicine medium, validation of care, likability of the telemedicine doctor, and willingness to be seen again by the telemedicine doctor. The fourth thematic category, *technology utilization* (Questions 2 and 3), is used to capture satisfaction with the patients' ability to hear and see the telemedicine doctor. The final category, *treatment access* (Questions 12, 13, and 16), was included to understand barriers to treatment as it relates to the time it took to obtain a tMOUD appointment, as well as the likeliness that the patient would have sought MOUD treatment had telemedicine not been available to them.

### Setting

Life's Energy Wellness Center, Inc. (LEWC Inc.), with three locations on Maryland's Eastern Shore, was utilized for the study setting to pilot this satisfaction instrument. The LEWC clinic is registered as a DEA clinic meeting criterion to prescribe MOUD via telehealth without requiring a face-to-face encounter. LEWC Inc. offers a variety of counseling services including individual, group, and family counseling as well as counseling for individuals suffering from SUDs. Although mental health and counseling services are available for clients, the towns in which LEWC Inc. operates have a paucity of DEA-waivered providers. The telemedicine partnership with DART addresses this need, providing much-needed MOUD to LEWC Inc. patients.

### Participant Selection

Using a convenience sampling method, new clients enrolling into LEWC Inc.'s IOP program (*N* = 14) were asked to voluntarily complete the telemedicine satisfaction survey immediately after completing an initial consultation with a DART telemedicine provider. LEWC Inc. staff members approached each new intake and determined interest in participation by asking “*Would you mind filling out a 5-min survey on your telemedicine experience? This is anonymous and will only be used for program quality assurance*.” Patients were assured that their involvement in the research survey would not interfere with the normal routine of clinical care received at the treatment center. Willing participants were given a touch screen tablet with an online survey link. Patients under 18 years of age and those already receiving treatment in the facility with telemedicine services were not invited to participate in the survey.

### Study Design

Our anonymous survey was administered via touchscreen tablet and data were electronically recorded into a REDCap database (43). The scoring system utilized Likert scales with responses ranging from 0 to 4, with numbers corresponding to responses of “strongly disagree” (a score of 0) to “strongly agree” (a score of 4). Because this was a limited pilot and proof-of-concept study, we limited data collection to occur only within 1 month, from September to October 2019.

### Results

We did not collect any personally identifying information from subjects who participated in our survey. Furthermore, as this study was conducted remotely as an anonymous, quality improvement (QI) program of evaluation, there were no direct interactions between the researchers and participants in the survey. Thus, this project was administratively reviewed by the University of Maryland, Baltimore Human Research Protections Office and was determined to not constitute research with human subjects. To characterize our patient sample we report demographics of all patients receiving tMOUD at the partnering site at which we collected survey data (*N* = 65; Table 1). Briefly, LEWC Inc.'s tMOUD patient population is predominantly White (76.9%) and predominantly male (56.9%). The mean age of patients enrolled in our tMOUD program is 35.6 years old (range = 19–66). tMOUD patients were more likely to have public insurance coverage (93.8%) and be prescribed buprenorphine (80%) as compared to naltrexone (20%). See Table 1 for full LEWC Inc. DART tMOUD program demographic data.

**Table 1:** Demographics of patients receiving treatment at LEWC Inc. enrolled in the Telemedicine with MOUD partnership with the University of Maryland School of Medicine.

**Baseline Characteristic**	***N***	**%**
**(*N* = 65)**		
Age at intake (years)		
Mean (±S.E.M.)	35.6 (±1.24)	
Range	19–66	
Gender		
Male	37	56.9
Female	28	43.1
Race		
White	50	76.9
Black	9	13.8
Other	4	6.2
Missing	2	3.1
Insurance Coverage		
Medicaid	61	93.8
Private	2	3.1
Self-Pay	2	3.1
Medication Type		
Buprenorphine	52	80
Naltrexone	13	20

A total of 14 new intake participants were approached, all of whom agreed to complete the tMOUD patient satisfaction survey. The preliminary findings showed an overall positive experience with tMOUD. Results of specific thematic categories are discussed below.

- *Communication*
  100% of respondents agreed or strongly agreed with the following statements:
  I could talk comfortably with the telemedicine doctor on the screen.
  It was easy to talk with the telemedicine doctor over the screen.
  I could talk about my problems easily.
  I understood the recommendations and know what the telemedicine doctor wants me to do.
  I felt like I was part of decisions made related to my screen care.
- *Privacy*
  64% of respondents disagreed with the statement “I was worried about others hearing me.”
- *Patient Perceptions*
  93% of respondents agreed or strongly agreed with the statement “I feel OK about the doctor's advice.”
  86% of respondents agreed or strongly agreed with the statement “I think other people would like the telemedicine doctor on the screen.”
  100% of respondents agreed or strongly agreed with the statement “I am willing to go back to this telemedicine doctor on the screen.”
  93% of respondents agreed or strongly agreed “I think that getting help over the screen was as good as getting help in person.”
  100% of respondents agreed or strongly agreed with the statement “I feel the amount of time spent during my telemedicine doctor visit was appropriate for my treatment needs.”
- *Technology Utilization*
  100% of respondents agreed or strongly agreed with the following statements:
  I could see the telemedicine doctor on the screen really well.
  I could hear the telemedicine doctor on the screen really well.
- *Treatment Access*
  36% of respondents agreed or strongly agreed with the statement “I would not have received opioid treatment were it not for telemedicine doctor”, while 29% reported a neutral response to this question, and 21% respondents disagreed or strongly disagreed.
  71% of respondents agreed or strongly agreed with the statement “The number of days waiting to see the telemedicine doctor for medication was reasonable.”
  79% of respondents agreed or strongly agreed with the statement “Following my initial assessment, I am satisfied with the amount of time it took for me to have an appointment with the telemedicine doctor.”

### Discussion

Telemedicine as a medium to deliver OUD treatment services is a feasible option for addressing the lack of access to MOUD in rural areas. Moreover, telemedicine is uniquely capable of addressing other barriers to accessing mental healthcare unrelated to geographic proximity to providers, such as stigma (the shame of addiction may preclude treatment) and anxiety surrounding an in-person encounter with a live doctor. These barriers aside, telemedicine has evolved with an increasingly technologically-inclined society. Indeed, when given the option, patients may even *prefer* telemedicine over routine in-person encounters with substance use disorder providers, as a recent report by Slightam et al. (44) suggests. This patient satisfaction survey serves as an important measure and provides guidance in assessing the satisfaction of patients regarding the quality of care provided via telemedicine. These pilot data show favorable satisfaction and acceptance by patients receiving tMOUD. Larger studies should further assess satisfaction to improve the way in which services are provided and delivered to patients.

It is important to note that these data were collected prior to the COVID-19 pandemic, and prior to the SAMHSA and DEA regulation changes to MOUD treatment. Along with others (45, 46), we (47) have argued against a full return to the pre-COVID *status quo* of restrictive, stigmatizing OUD treatment regulations. Further, we strongly encourage researchers to take advantage of the natural experiment availed by the pandemic to assess how a less restrictive environment might affect treatment outcomes (47). Part of these research efforts should include patient perspectives, as patient satisfaction will ultimately determine uptake and implementation of telemedicine in an OUD treatment practice.

A major limitation of our study is its small sample size. Although we were encouraged by the high rate of engagement (of the 14 patients approached, all 14 agreed to take part in the survey), the administration of the survey was dependent on LEWC Inc. staff involvement and time. Intake consultations can be cumbersome to both staff and patients, and clinical treatment needs would have taken precedence over completion of a voluntary survey.

Another limitation is that our assessment queried patient satisfaction at only one time, immediately following the initial telemedicine evaluation. Because this study aimed to determine whether the tMOUD platform was an acceptable medium for rural patients receiving MOUD, and was conducted as a QI project, we did not conduct follow-up assessments with patients to explore their satisfaction over the course of their treatment. Future research efforts will aim to assess satisfaction at multiple time points across treatment trajectory, particularly important considering that satisfaction may change over time, which may impact treatment engagement and retention.

A third limitation is that these pilot data utilized a convenience sampling method and did not include any randomization or control groups. Provider variation may impact the patient experience. As a proof-of-concept evaluation, we did not control for the number of telemedicine providers, nor did we balance the telemedicine encounters across our clinicians—one factor among several that could greatly impact patient satisfaction.

Finally, it is worth noting that although this instrument was derived using an existing body of literature that based global patient satisfaction with telemedicine on five themes, our particular instrument was not subjected to the rigorous psychometric testing that would be necessary for field adoption. The select rural population in which this survey was conducted is situated in one eastern county of the state of Maryland; other rural areas of Maryland may have different experiences. Prior to its implementation, this survey would need to undergo tests of reliability, validation and generalizability. Future studies will address these limitations.

### Future Directions

As a growing number of rural communities and correctional institutions recognize the value in providing OUD treatment via telemedicine, evaluations of satisfaction will be needed as telemedicine programs are developed and expanded. A greater understanding of patient satisfaction with tMOUD will also help to inform the integration of mobile phone or app-based platforms, which may help to mitigate transportation barriers in rural areas. Assessing satisfaction in treatment over time is important given the high rates of dropout for rural MOUD patients who are prescribed buprenorphine (48).

Additionally, provider satisfaction should continue to be assessed to ensure clinicians' validation of evolving telemedicine mediums. Future studies should conduct psychometric evaluations of this instrument to ensure consistency, validity and generalizability to be able to conduct the necessary refinements that would allow for longitudinal studies of patient satisfaction. Finally, research efforts will assess whether this instrument may have prospective predictive value for treatment engagement or retention.

## Data Availability Statement

The raw data supporting the conclusions of this article will be made available by the authors, without undue reservation.

## Ethics Statement

The studies involving human participants were reviewed and approved by Institutional Review Board, University of Maryland, Baltimore. Written informed consent for participation was not required for this study in accordance with the national legislation and the institutional requirements.

## Author Contributions

All authors contributed to the conception of this paper. AB developed the patient satisfaction survey. TC and DR conducted the literature search. TC wrote the first draft of the manuscript. All authors provided critical feedback, revisions to the manuscript, and approved the submitted version.

## Conflict of Interest

AK-F was employed by the company Life's Energy Wellness Center, Inc. The remaining authors declare that the research was conducted in the absence of any commercial or financial relationships that could be construed as a potential conflict of interest.

## References

[1] Hancock C Mennenga H King N Andrilla H Larson E Schou P Treating the Rural Opioid Epidemic. National Rural Health Association (2017). Available online at: https://www.ruralhealthweb.org/NRHA/media/Emerge_NRHA/Advocacy/Policy%20documents/2019-NRHA-Policy-Document-Treating-the-Rural-Opioid-Epidemic.pdf (accessed April 3, 2020).

[2] Corso C Townley C Intervention, Treatment, and Prevention Strategies to Address Opioid Use Disorders in Rural Areas: A Primer on Opportunities For Medicaid-Safety Net Collaboration. (2016). Available online at: https://nashp.org/wp-content/uploads/2016/09/Rural-Opioid-Primer1.pdf (accessed April 3, 2020).

[3] Shigekawa E Fix M Corbett G Roby DH Coffman J. The current state of telehealth evidence: a rapid review. Health Aff (Millwood). (2018) 37:1975–82. 10.1377/hlthaff.2018.05132 30633674

[4] Dick AW Pacula RL Gordon AJ Sorbero M Burns RM Leslie D. Growth in buprenorphine waivers for physicians increased potential access to opioid agonist treatment, 2002–11. Health Aff (Millwood). (2015) 34:1028–34. 10.1377/hlthaff.2014.1205 26056209 PMC4743254

[5] Rosenblatt RA Andrilla CHA Catlin M Larson EH. Geographic and specialty distribution of US physicians trained to treat opioid use disorder. Ann Fam Med. (2015) 13:23–6. 10.1370/afm.1735 25583888 PMC4291261

[6] Centers for Medicare & Medicaid Services (CMS) Telemedicine. (2015). Available online at: https://www.medicaid.gov/medicaid/benefits/telemedicine/index.html (accessed April 3, 2020).

[7] Hawkins EJ Lott AMK Malte CA Frank AN Hamilton B Sayre GG. Patients' perspectives on care management services for complex substance use disorders. J Addict Dis. (2017) 36:193–206. 10.1080/10550887.2017.1326654 28481144 PMC9870041

[8] Barbosa CD Balp M-M Kulich K Germain N Rofail D. A literature review to explore the link between treatment satisfaction and adherence, compliance, and persistence. Patient Prefer Adherence. (2012) 6:39–48. 10.2147/PPA.S24752 22272068 PMC3262489

[9] Kelly SM O'Grady KE Brown BS Mitchell SG Schwartz RP. The role of patient satisfaction in methadone treatment. Am J Drug Alcohol Abuse. (2010) 36:150–4. 10.3109/00952991003736371 20465372 PMC2938880

[10] Hser Y-I Evans E Huang D Anglin DM. Relationship between drug treatment services, retention, and outcomes. Psychiatr Serv. (2004) 55:767–74. 10.1176/appi.ps.55.7.767 15232015

[11] Hedegaard H Miniño AM Warner M. Urban–Rural Differences in Drug Overdose Death Rates, by Sex, Age, and Type of Drugs Involved, 2017. Center for Disease Control and Prevention. (2019). Available online at: https://stacks.cdc.gov/view/cdc/80469 (accessed April 3, 2020). 31442197

[12] Lister JJ Weaver A Ellis JD Himle JA Ledgerwood DM. A systematic review of rural-specific barriers to medication treatment for opioid use disorder in the United States. Am J Drug Alcohol Abuse. (2020) 46:273–88. 10.1080/00952990.2019.1694536 31809217

[13] Kelly JF Humphreys K Ferri M. Alcoholics Anonymous and other 12-step programs for alcohol use disorder. Cochrane Database Syst Rev. (2020) 3:CD012880. 10.1002/14651858.CD012880.pub2 32159228 PMC7065341

[14] Mattick RP Breen C Kimber J Davoli M. Methadone maintenance therapy versus no opioid replacement therapy for opioid dependence. Cochrane Database Syst Rev. (2009) 2009:CD002209. 10.1002/14651858.CD002209.pub2 12519570

[15] Volkow ND Frieden TR Hyde PS Cha SS. Medication-assisted therapies — tackling the opioid-overdose epidemic. N Engl J Med. (2014) 370:2063–6. 10.1056/NEJMp1402780 24758595

[16] Connery HS. Medication-assisted treatment of opioid use disorder: review of the evidence and future directions. Harv Rev Psychiatry. (2015) 23:63–75. 10.1097/HRP.0000000000000075 25747920

[17] Andrilla CHA Moore TE Patterson DG Larson EH. Geographic distribution of providers with a dea waiver to prescribe buprenorphine for the treatment of opioid use disorder: a 5-year update. J Rural Health. (2019) 35:108–12. 10.1111/jrh.12307 29923637

[18] Garnick DW Horgan CM Acevedo A Lee MT Panas L Ritter GA. rural clients' continuity into follow-up substance use disorder treatment: impacts of travel time, incentives, and alerts. J Rural Health. (2020) 36:196–207. 10.1111/jrh.12375 31090968 PMC6856385

[19] Rosenblum A Cleland CM Fong C Kayman DJ Tempalski B Parrino M. Distance traveled and cross-state commuting to opioid treatment programs in the United States. J Environ Public Health. (2011) 2011:948789. 10.1155/2011/948789 21776440 PMC3136171

[20] Rigg KK Monnat SM Chavez MN. Opioid-related mortality in rural America: geographic heterogeneity and intervention strategies. Int J Drug Policy. (2018) 57:119–29. 10.1016/j.drugpo.2018.04.011 29754032

[21] Keyes KM Cerdá M Brady JE Havens JR Galea S. Understanding the rural–urban differences in nonmedical prescription opioid use and abuse in the United States. Am J Public Health. (2014) 104:e52–9. 10.2105/AJPH.2013.301709 24328642 PMC3935688

[22] Rigg KK Monnat SM. Urban vs. rural differences in prescription opioid misuse among adults in the United States: informing region specific drug policies and interventions. Int J Drug Policy. (2015) 26:484–91. 10.1016/j.drugpo.2014.10.001 25458403 PMC4397122

[23] American Psychiatric Association (APA) What is Telepsychiatry? (2017). Available online at: https://www.psychiatry.org/patients-families/what-is-telepsychiatry (accessed April 3, 2020).

[24] American Society of Addiction Medicine (ASAM) COVID-19 Legislation: Summary of Provisions for Addiction Medicine. (2020). Available online at: https://www.asam.org/advocacy/news/asam-advocacy-blog/2020/03/26/covid-19-legislation-summary-of-provisions-for-addiction-medicine (accessed April 3, 2020).

[25] Weintraub E Greenblatt AD Chang J Welsh CJ Berthiaume AP Goodwin SR Outcomes for patients receiving telemedicine-delivered medication-based treatment for opioid use disorder: a retrospective chart review. Heroin Addict Relat Clin Probl. (2020). [Epub ahead of print]. PMC7861202 33551692

[26] Weintraub E Greenblatt AD Chang J Himelhoch S Welsh C. Expanding access to buprenorphine treatment in rural areas with the use of telemedicine: buprenorphine in rural areas with telemedicine. Am J Addict. (2018) 27:612–7. 10.1111/ajad.12805 30265425

[27] Zheng W Nickasch M Lander L Wen S Xiao M Marshalek P. Treatment outcome comparison between telepsychiatry and face-to-face buprenorphine Medication-Assisted Treatment (MAT) for opioid use disorder: a 2-year retrospective data analysis. J Addict Med. (2017) 11:138–44. 10.1097/ADM.0000000000000287 28107210 PMC5354971

[28] Maryland Opioid Operational Command Center (MDOOCC) Annual Report January 1, 2019- December 31, 2019. Office of the Governor (2020). Available online at: https://health.maryland.gov/vsa/Documents/Overdose/Annual_2019_Drug_Intox_Report.pdf (accessed April 3, 2020).

[29] FORE Our Grantees. Foundation for Opioid Response Efforts (FORE) (2020). Available online at: https://forefdn.org/our-grantees/ (accessed April 3, 2020).

[30] Drug Enforcement Administration (DEA) Department of Justice Implementation of the Ryan Haight Online Pharmacy Consumer Protection Act of 2008. Interim final rule with request for comments. Fed Regist. (2009) 74:15595–625. 19507319

[31] Alexander GC Stoller KB Haffajee RL Saloner B. An Epidemic in the midst of a pandemic: opioid use disorder and COVID-19. Ann Intern Med. (2020) 173:57–8. 10.7326/M20-1141 32240283 PMC7138407

[32] Drug Enforcement Administration Drug Enforcement Administration. Letter to DEA Qualifying Practitioners. (2020). Available online at: https://www.samhsa.gov/sites/default/files/dea-samhsa-buprenorphine-telemedicine.pdf?mc_cid=8dffbfc637&mc_eid=d4494a732e (accessed April 3, 2020).

[33] Carlson MJ Gabriel RM. Patient satisfaction, use of services, and one-year outcomes in publicly funded substance abuse treatment. Psychiatr Serv. (2001) 52:1230–6. 10.1176/appi.ps.52.9.1230 11533398

[34] Lin L (Allison) Casteel D Shigekawa E Weyrich MS Roby DH McMenamin SB. Telemedicine-delivered treatment interventions for substance use disorders: a systematic review. J Subst Abuse Treat. (2019) 101:38–49. 10.1016/j.jsat.2019.03.007 31006553

[35] Eibl JK Gauthier G Pellegrini D Daiter J Varenbut M Hogenbirk JC. The effectiveness of telemedicine-delivered opioid agonist therapy in a supervised clinical setting. Drug Alcohol Depend. (2017) 176:133–8. 10.1016/j.drugalcdep.2017.01.048 28535455

[36] Bruyneel L Tambuyzer E Coeckelberghs E Wachter DD Sermeus W Ridder DD. New instrument to measure hospital patient experiences in Flanders. Int J Environ Res Public Health. (2017) 14:1319. 10.3390/ijerph14111319 29084160 PMC5707958

[37] Tarp K Mejldal A Nielsen AS. Patient satisfaction with videoconferencing-based treatment for alcohol use disorders. Addict Disord Their Treat. (2017) 16:70–9. 10.1097/ADT.0000000000000103 28553192 PMC5434970

[38] Morgan RD Patrick AR Magaletta PR. Does the use of telemental health alter the treatment experience? Inmates' perceptions of telemental health versus face-to-face treatment modalities. J Consult Clin Psychol. (2008) 76:158–62. 10.1037/0022-006X.76.1.158 18229993

[39] Hilty DM Nesbitt TS Kuenneth CA Cruz GM Hales RE. Rural versus suburban primary care needs, utilization, and satisfaction with telepsychiatric consultation. J Rural Health. (2007) 23:163–5. 10.1111/j.1748-0361.2007.00084.x 17397373

[40] Frueh BC Henderson S Myrick H. Telehealth service delivery for persons with alcoholism. J Telemed Telecare. (2005) 11:372–5. 10.1177/1357633X0501100701 16238840

[41] Bishop JE O'Reilly RL Maddox K Hutchinson LJ. Client satisfaction in a feasibility study comparing face-to-face interviews with telepsychiatry. J Telemed Telecare. (2002) 8:217–21. 10.1258/135763302320272185 12217104

[42] Brodey BB Claypoole KH Motto J Arias RG Goss R. Satisfaction of forensic psychiatric patients with remote telepsychiatric evaluation. Psychiatr Serv. (2000) 51:1305–7. 10.1176/appi.ps.51.10.1305 11013332

[43] Harris PA Taylor R Thielke R Payne J Gonzalez N Conde JG. Research electronic data capture (REDCap)—A metadata-driven methodology and workflow process for providing translational research informatics support. J Biomed Inform. (2009) 42:377–81. 10.1016/j.jbi.2008.08.010 18929686 PMC2700030

[44] Slightam C Gregory AJ Hu J Jacobs J Gurmessa T Kimerling R. Patient perceptions of video visits using veterans affairs telehealth tablets: survey study. J Med Internet Res. (2020) 22:e15682. 10.2196/15682 32293573 PMC7191342

[45] Davis CS Samuels EA. Opioid policy changes during the COVID-19 pandemic - and beyond. J Addict Med. (2020) 14:e4–5. 10.1097/ADM.0000000000000679 32433363 PMC7273953

[46] Del Pozo B Beletsky L Rich JD. COVID-19 as a frying pan: the promise and perils of pandemic-driven reform. J Addict Med. (2020) 14:e144–6. 10.1097/ADM.0000000000000703 32604133 PMC9204673

[47] Greenblatt AD Magidson JF Belcher AM Gandhi D Weintraub E. Overdue for an overhaul: how opioid treatment programs can learn from COVID-19. Mayo Clin Proc. (2020) 95:2076–8. 10.1016/j.mayocp.2020.08.011 33012340 PMC7447258

[48] Saloner B Daubresse M Alexander GC. Patterns of buprenorphine-naloxone treatment for opioid use disorder in a multi-state population. Med Care. (2017) 55:669–76. 10.1097/MLR.0000000000000727 28410339 PMC6528471

