# Supplementary material for: Patient Satisfaction With Medications for Opioid Use Disorder Treatment via Telemedicine: Brief Literature Review and Development of a New Assessment
Source: Front Public Health. 2021 Jan 21;8:557275. doi: 10.3389/fpubh.2020.557275 (PMC7859509; doi:10.3389/fpubh.2020.557275)
Supplement: Supplementary file 1 [file Data_Sheet_1.pdf]

Appendix 1

Patient Satisfaction Survey

1. I could talk comfortably with the telemedicine doctor on the screen. *(i)*
2. I could see the telemedicine doctor on the screen really well. *(iv)*
3. I could hear the telemedicine doctor on the screen really well. *(iv)*
4. I was worried about others hearing me. *(ii)*
5. It was easy to talk with the telemedicine doctor over the screen. *(i)*
6. I could talk about my problem easily. *(i)*
7. I understood the recommendation and know what the telemedicine doctor wants me to do. *(i)*
8. I feel OK about the doctor’s advice. *(iii)*
9. I think other people would like the telemedicine doctor on the screen. *(iii)*
10. I am willing to go back to this telemedicine doctor on the screen. *(iii)*
11. I think that getting help over the screen was as good as getting help in person. *(iii)*
12. I would not have received opioid treatment were it not for telemedicine doctor. *(v)*
13. The number of days waiting to see the telemedicine doctor for medication was reasonable. *(v)*
14. I feel the amount of time spent during my telemedicine doctor visit was appropriate for my treatment needs. *(iii)*
15. I felt like I was a part of decisions made related to my screen care. *(i)*
16. Following my initial assessment, I am satisfied with the amount of time it took for me to have an appointment with the telemedicine doctor. *(v)*

Key to thematic categories: (i) Communication; (ii) Privacy; (iii) Patient Perceptions; (iv) Technology Utilization; (v) Treatment Access
